# Age Differences in the Association Between Anxiety Symptoms and Suicide Risk

**DOI:** 10.1093/geroni/igab046.2019

**Published:** 2021-12-17

**Authors:** Montgomery Owsiany, Ruifeng Cui, Amy Fiske

**Affiliations:** 1 West Virginia University, Morgantown, West Virginia, United States; 2 VA Pittsburgh Healthcare System, Pittsburgh, Pennsylvania, United States

## Abstract

Suicide rates increase over the life-span, necessitating concern in older adults. Recent studies suggest that anxiety disorders are associated with suicidal thoughts and behavior. The present study examined the association between anxiety symptoms (General Anxiety Disorder-7) and suicide risk (Suicide Behaviors Questionnaire-Revised), testing whether the association differs between younger and older adults. Depression symptoms (Patient Health Questionnaire-8) were controlled for in the analyses. In a sample of 944 participants (46% 60+ years), anxiety symptoms, depression symptoms, and suicide risk were lower among older adults (60+ years) than younger adults (all p < .01). Age moderated the significant association between anxiety symptoms and suicide risk (ΔR2 = .008, p < .01). Results indicate that an increase in anxiety is associated with a smaller increase in suicide risk for older adults than younger adults. The need for suicide risk screening among individuals with elevated anxiety symptoms is critical, especially for younger adults.

